# Do interindividual differences in cardiac output during submaximal exercise explain differences in exercising muscle oxygenation and ratings of perceived exertion?

**DOI:** 10.14814/phy2.13570

**Published:** 2018-01-25

**Authors:** Robert F. Bentley, Joshua H. Jones, Daniel M. Hirai, Joel T. Zelt, Matthew D. Giles, James P. Raleigh, Joe Quadrilatero, Brendon J. Gurd, J. Alberto Neder, Michael E. Tschakovsky

**Affiliations:** ^1^ Human Vascular Control Laboratory School of Kinesiology and Health Studies Queen's University Kingston Ontario Canada; ^2^ Laboratory of Clinical Exercise Physiology Division of Respirology Department of Medicine Queen's University Kingston Ontario Canada; ^3^ Queen's Muscle Physiology Laboratory School of Kinesiology and Health Studies Queen's University Kingston Ontario Canada; ^4^ Muscle Biology and Cell Death Laboratory Department of Kinesiology University of Waterloo Waterloo Ontario Canada

**Keywords:** Cardiac output, exercise tolerance, interindividual differences, ratings of perceived exertion

## Abstract

Considerable interindividual differences in the $\dot{Q}-\dot{V}O_{2}$ relationship during exercise have been documented but implications for submaximal exercise tolerance have not been considered. We tested the hypothesis that these interindividual differences were associated with differences in exercising muscle deoxygenation and ratings of perceived exertion (RPE) across a range of submaximal exercise intensities. A total of 31 (21 ± 3 years) healthy recreationally active males performed an incremental exercise test to exhaustion 24 h following a resting muscle biopsy. Cardiac output ($\dot{Q}$ L/min; inert gas rebreathe), oxygen uptake ($\dot{V}O_{2}$ L/min; breath‐by‐breath pulmonary gas exchange), quadriceps saturation (near infrared spectroscopy) and exercise tolerance (6–20; Borg Scale RPE) were measured. The $\dot{Q}-\dot{V}O_{2}$ relationship from 40 to 160 W was used to partition individuals post hoc into higher (*n* = 10; 6.3 ± 0.4) versus lower (*n* = 10; 3.7 ± 0.4, *P* < 0.001) responders. The $\dot{Q}-\dot{V}O_{2}$ difference between responder types was not explained by arterial oxygen content differences (*P* = 0.5) or peripheral skeletal muscle characteristics (*P* from 0.1 to 0.8) but was strongly associated with stroke volume (*P* < 0.05). Despite considerable $\dot{Q}-\dot{V}O_{2}$ difference between groups, no difference in quadriceps deoxygenation was observed during exercise (all *P* > 0.4). Lower cardiac responders had greater leg (*P* = 0.027) and whole body (*P* = 0.03) RPE only at 185 W, but this represented a higher %peak $\dot{V}O_{2}$ in lower cardiac responders (87 ± 15% vs. 66 ± 12%, *P* = 0.005). Substantially lower $\dot{Q}-\dot{V}O_{2}$ in the lower responder group did not result in altered RPE or exercising muscle deoxygenation. This suggests substantial recruitment of blood flow redistribution in the lower responder group as part of protecting matching of exercising muscle oxygen delivery to demand.

## Introduction

Systemic oxygen delivery (cardiac output; $\dot{Q}$) increases in proportion to exercising muscle oxygen consumption ($\dot{V}O_{2}$) and is closely coupled to the increase in exercising muscle blood flow (eMBF) (Mortensen et al. 2008; Calbet et al. 2015). A potential modifier of this coupling is the contribution of vasoconstriction in nonexercising muscle tissue, which could allow eMBF to increase more than $\dot{Q}$ due to redistribution of peripheral perfusion as demonstrated in animal studies (Armstrong et al. 1987). Arterial blood pressure results from the interaction of $\dot{Q}$ and the total vascular conductance (TVC) and also requires regulation during exercise hyperemia. A greater $\dot{Q}$ increase for any given submaximal exercising muscle oxygen demand would allow for the regulation of arterial blood pressure at a higher exercising muscle vascular conductance, with subsequently greater exercising muscle oxygen delivery (O_2_D).

At present, the $\dot{Q}$ response to an increase in oxygen demand presents with a “typical” slope of ~5–7 L/min of $\dot{Q}$ per 1 L/min of $\dot{V}O_{2}$ (Faulkner et al. 1977; Proctor et al. 1998; Adami et al. 2014). However, there is evidence for considerable interindividual variation at the same submaximal $\dot{V}O_{2}$ with $\dot{Q}$ differences of up to ~5 L/min being observed (Reeves et al. 1961; Astrand et al. 1964; Yamaguchi et al. 1986). Within an individual, increases in submaximal $\dot{Q}$ arise following hemodilution (Roach et al. 1999), while the slope of $\dot{Q}-\dot{V}O_{2}$ is increased in hypoxia (Adami et al. 2014). These within subject findings have been assumed to apply to interindividual differences in $\dot{Q}$ (Faulkner et al. 1977, Proctor et al. 1998; Adami et al. 2014).

To the best of our knowledge, only one study has actually sought to interrogate interindividual differences in submaximal $\dot{Q}$ (Freedson 1981). During submaximal cycling exercise in women, it was revealed that [Hb] between ~10‐13.5 g/100 mL blood was negatively correlated with $\dot{Q}$. [Hb] greater than 13.5 g/100 mL blood appear to have no effect on $\dot{Q}$. While C_a_O_2_ was not reported in the previously mentioned investigations (Reeves et al. 1961; Astrand et al. 1964; Yamaguchi et al. 1986), the findings of (Freedson 1981), regarding a threshold [Hb] at ~13.5 g/100 mL blood above which there is no effect on interindividual differences in $\dot{Q}-\dot{V}O_{2}$, would argue against the magnitude of interindividual differences in $\dot{Q}-\dot{V}O_{2}$ in males simply reflecting compensation for C_a_O_2_ differences between individuals. Given the well‐established effect of altered O_2_D within an individual on exercise tolerance (for review see, Hepple 2002), the potential impact of interindividual differences in $\dot{Q}-\dot{V}O_{2}$ on oxygen supply to submaximal exercising muscle warrants consideration.

An individual's ratings of perceived exertion (RPE) (verbal expression of how “hard” exercise feels on the Borg Scale (Borg 1970)) provides a representation of the psychophysiological stress of exercise (Abbiss et al. 2015). During exercise, RPE is reduced in hyperoxia (Eves et al. 2002), increased in hypoxia (MacNutt et al. 2015) and increased in response to fatigue (Eiken and Bjurstedt 1987). Within individuals these investigations speak to the effects of manipulating O_2_D and the sensitivity of RPE to cellular homeostasis. However, they fail to address the implications of potential interindividual differences in $\dot{Q}-\dot{V}O_{2}$ on exercise tolerance as represented by RPE.

Therefore, the purpose of this study was to determine whether interindividual differences in $\dot{Q}-\dot{V}O_{2}$ during exercise in men were associated with differences in exercising muscle deoxygenation and RPE across a range of submaximal exercise intensities. Given that variation in skeletal muscle capillary density (Brodal et al. 1977; Zoladz et al. 2005) and therefore diffusive conductance, coupled with individual variability in mitochondrial enzymatic activity (Zurlo et al. 1994) could impact metabolic and contractile function at a given convective O_2_D, we also undertook measurements of these characteristics to account for their potential influence on the $\dot{Q}-\dot{V}O_{2}$ relationship. We hypothesized that healthy young men would differ considerably in their cardiac response during exercise and that a lower $\dot{Q}-\dot{V}O_{2}$ would be associated with reduced muscle oxygenation and increased RPE at a given submaximal exercise intensity.

## Methods

### Participants

Thirty‐one healthy, recreationally active (<3 h/week of structured exercise) male participants (21 ± 3 years) with no history of smoking, cardiovascular disease or hypertension participated. Participants for all intent and purposes were a homogeneous male subset from our population of interest. This study was approved by the Health Sciences Research Ethics Board at Queen's University according to the terms of the Declaration of Helsinki. Procedures were in accordance with institutional guidelines. Each participant provided signed consent after receiving complete verbal and written descriptions of the experimental protocol and potential risks.

### Experimental design

**Figure 1:**
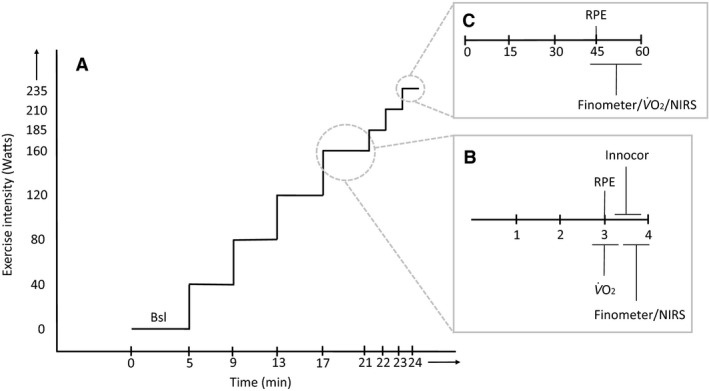
Progressive exercise test and measurement timing. (A) Progressive exercise test beginning with rest and increasing by 40 W every 4 min until 160 W. Beyond 160 W, exercise increased by 25 W every minute until volitional exhaustion. (B) Timing of measurements (in minutes) during exercise up to 160 W. (C) Timing of measurements (in seconds) during exercise beyond 160 W. RPE, rating of perceived exertion; $\dot{V}O_{2}$, rate of oxygen consumption; NIRS, near infrared spectroscopy.

This was a between participant design in which each participant completed 2 visits to the laboratory. On the first visit, a venous blood sample and a muscle biopsy (details to follow) were obtained. On the second visit, one incremental cycling exercise test to volitional exhaustion was completed. A single exercise test was completed due to the strong reproducibly in the assessment of $\dot{Q}$ and $\dot{V}O_{2}$. Prior bouts of submaximal cycling demonstrated a within participant between day coefficient of variation of 2.2% and 2.8%, respectively (*n* = 4). Given the low level of variability, modified Monte Carlo simulations determined that multiple repeats were not required to state with confidence that a particular individual had a different cardiac response from someone else (Fig. 1).

### Incremental exercise test

The day after the venous blood sample and resting muscle biopsy, participants arrived at Kingston General Hospital for a progressive exercise test to exhaustion. Each participant completed a progressive cycling exercise on an electronically braked cycle ergometer (VIAsprint 150P; Ergoline, Bitz, Germany). Seat height was adjusted for each participant to a level deemed comfortable by each participant. Participants rested on the bike for 5 min. Exercise started at 40 W (at a self‐selected rpm) and increased every 4 min until 160 W. Following 160 W, exercise intensity increased by 25 W every minute until volitional exhaustion. During the exercise systemic blood pressure, $\dot{Q}$, $\dot{V}O_{2}$ and RPE were measured.

### Instrumentation and data acquisition

#### Standard anthropometric data

Upon arrival in the laboratory, standard anthropometric data was obtained for each subject. Age, height, weight and waist circumference were obtained. A 7‐day physical activity recall adapted from (Sarkin et al. 1997) was completed to quantify current exercise habits.

#### Venous blood sample and assessment of skeletal muscle properties

Participants arrived to the lab in the morning after ~8 h of fasting. Upon arrival at the lab, a supine resting blood sample was obtained using standard venipuncture into a 4.5 mL lithium heparin vacutainer. The tube was inverted 8–10 times and then immediately analyzed for hemoglobin content with a blood gas analyzer. Following the blood sample, participants underwent a resting muscle biopsy using the Bergstrom needle biopsy technique (Bergstrom 1975). Biopsies were performed under sterile conditions with local anesthesia (2% lidocaine) using a custom‐modified Bergstrom biopsy needle and manually applied suction. Following the biopsy, muscle tissue was immediately blotted, snap‐frozen in liquid nitrogen, and stored at −80°C until analyzed.

#### Cardiac output – inert gas rebreathe

In a five‐breath maneuver, participants breathed a mixture of three gases (99.4% oxygen, 0.5% nitrous oxide, and 0.1% sulfur hexafluoride) from a rebreathing bag (Innocor, Innovision, GbH). Briefly, sulfur hexafluoride is insoluble and is used to calculate lung volume. Nitrous oxide is a soluble gas that is absorbed by the blood, and is harmless at these concentrations during exercise with 2–3 min between tests. The disappearance of nitrous oxide into the blood allows for the measurement of pulmonary blood flow and therefore $\dot{Q}$ in healthy persons. Breathing maneuvers were completed at rest and during exercise at 40 W increments once every 4 min up to 160 W. Maneuvers were implemented after controlling for breathing frequency and tidal volume and occurred during steady state. This rebreathing method correlates well with direct Fick measures of $\dot{Q}$ *r* = 0.94 (Agostoni et al. 2005) with a mean bias of +0.34 ± 0.59 L/min (Peyton and Thompson 2004) and demonstrates strong reproducibility (coefficient of variation 4.3%) during submaximal exercise in healthy participants (Fontana et al. 2009). Pilot work (*n* = 4) demonstrated that our $\dot{Q}$ variability (assessed both within and between day) was 2.2% for exercise intensities up to 200 W.

#### Cardiac output – finger photoplethysmography

A finger photoplethysmograph was placed on the middle finger of the left hand to measure mean arterial blood pressure throughout exercise. Both arms were supported by clip‐on Bontrager aerobars (Trek, Waterloo, WI) and therefore the hand remained in the same position relative to heart level at all times. The aerobars also allowed for the hands to remain relaxed throughout exercise. In addition to blood pressure, this device provides estimates of stroke volume (SV) and thereby computed $\dot{Q}$ and total peripheral resistance via the ModelFlow™ (Finapres Medical Systems, the Netherlands).

#### Quadriceps muscle oxygenation status

Quadriceps oxygenation of the right leg was measured by frequency‐domain multidistance near‐infrared spectroscopy (FDMD NIRS, Imagent; ISS, Champaign, IL). Principles of operation and algorithms have been described in detail previously (Gratton et al. 1997). The probe was secured to the skin over the vastus lateralis muscle ~14 cm above the patella with adhesive tape and an elastic bandage. FDMD NIRS provides measurements of the absolute concentrations of oxygenated and deoxygenated hemoglobin/myoglobin (oxy‐[Hb/Mb] and deoxy‐[Hb/Mb], respectively). Measures of total heme concentration (total [Hb/Mb] = oxy‐[Hb/Mb]+deoxy‐[Hb/Mb]) and tissue oxygen saturation (StO_2_ = oxy‐[Hb/Mb]/total [Hb/Mb], %) were also calculated. Oxygenation status of the muscle was recorded continuously throughout exercise.

#### Gas exchange and ventilation

Pulmonary gas exchange and ventilation were measured breath‐by‐breath using a calibrated, computer‐based system (Vmax Encore 229, CareFusion, Yorba Linda, CA). Both gas and volume calibrations were calibrated prior to each exercise testing session. Oxygen and carbon dioxide were calibrated using a two‐point calibration with atmospheric air and a known gas mixture (16% oxygen, 4% carbon dioxide). Volume calibration was completed with a 3 L volume syringe in accordance to the specifications outlined by the manufacture. Pulmonary gas exchange was measured continuously.

#### Heart rate and RPE

Participants were outfitted with a three lead electrocardiogram for the measurements of heart rate (HR) throughout exercise. Three minutes into rest and during steady state exercise from 40 to 160 W, ratings of whole body perceived exertion (RPE_WB_) and peripheral exertion of the legs (RPE_L_) were obtained in succession on the Borg 6–20 scale. Prior to exercise participants were oriented to the scale and read a script taken from (Faulkner and Eston 2007) to ensure correct understanding of exertional assessment. Beyond 160 W, RPE was obtained during the last 15 sec of an exercise intensity.

### Data analysis

#### Venous effluent – hemoglobin concentration

The venous blood sample was analyzed with a blood gas analyzer (Stat Profile Prime Blood Gas Analyzer, Nova Biomedical, Mississauga, Canada) for hemoglobin concentration.

#### Immunofluorescent and histochemical analysis

##### Skeletal muscle fiber type

Immunofluorescent analysis of myosin heavy chain (MHC) isoforms was performed as previously described (Bloemberg and Quadrilatero 2012; Scribbans et al. 2014) using primary antibodies against MHC I (BA‐F8), MHC IIa (SC‐71), and MHC IIx (6H1) (Developmental Studies Hybridoma Bank, Iowa City, IA). Sections were incubated with a primary antibody against dystrophin (MANDYS1 [3b7], Developmental Studies Hybridoma Bank) to identify the muscle membrane. Fiber types were identified by isotype‐specific fluorescent secondary antibodies (type I, blue; type IIa, green; type IIX, red; as well type IIAX hybrid fibers). For all immunofluorescent procedures, sections were mounted with Prolong Gold Antifade Reagent (Life Technologies, Burlington, Ontario, Canada) and imaged the following day. All sections were visualized with an Axio Observer Z1 microscope (Carl Zeiss, Jena, TH, Germany). Individual images were taken across the entire muscle cross‐section and assembled into a composite panoramic image using AxioVision software (Carl Zeiss).

##### Capillary density

Quantification of capillary density was completed as previously described (Scribbans et al. 2014). Briefly, sections of muscle tissue were fixed in 4% paraformaldehyde for 10 min, followed by permeabilization with 0.5% TritonX‐100 for 30 min, and then blocked in 10% goat serum for 30 min. Sections were then incubated overnight in 5% goat serum with the appropriate primary antibodies specific for the endothelium (PECAM) and sarcolemma (dystrophin) (Developmental Studies Hybridoma Bank, Iowa City, IA). After 3, 5 min washes in phosphate buffered saline, sections were incubated for 1 h in 5% goat serum with the appropriate fluorescent secondary antibodies (Life Technologies).

##### Succinate dehydrogenase activity

Histochemical staining for succinate dehydrogenase (SDH) activity was completed as previously described (Bloemberg and Quadrilatero 2012) to quantify mitochondrial content as this mitochondrial enzyme is strongly correlated with mitochondrial content (*r* = 0.73) (Larsen et al. 2012). A brightfield Nikon microscope linked to a PixeLink digital camera were was used to acquire images. Individual images were taken across the entire muscle cross‐section and assembled into a composite panoramic image using Microsoft Image Composite Editor (ICE) (Microsoft, Redmond, WA). Images were analyzed in ImageJ and calculated by subtracting background staining. Compiled images were matched to fiber‐type images and ~40 of each fiber type were randomly selected and analyzed. Data were expressed relative to the values obtained in type I and II fibers, which were assigned a reference value of 1.0, and reported as mean optical density in arbitrary units.

#### Oxygen consumption

Oxygen consumption was obtained on a breath‐by‐breath basis. At rest and up to 160 W, a 30 sec average was computed within the last 1 min and 30 sec of an exercise intensity. Beyond 160 W a 15 sec average was computed during the last 15 sec of a completed exercise intensity.

#### Cardiac output – inert gas rebreathe

Pulmonary blood flow (Cardiac Output; $\dot{Q}$) was measured during the last ~30 sec of rest and each completed exercise intensity up to 160 W. Beyond 160 W inert gas rebreathe was not completed due to: (1) the length of time to complete the test and the requirements for $\dot{V}O_{2}$ and RPE. (2) The manufacturer suggested washout period of 2 min during exercise. (3) The sudden cessation of exercise at exhaustion making peak assessments extremely challenging. Therefore, we utilized the following method below to obtain more accurate $\dot{Q}$ measures during higher intensity exercise.

#### Cardiac output – finger photoplethysmography

Finometer measures of arterial blood pressure and estimates of $\dot{Q}$ were obtained on a beat‐by‐beat basis. $\dot{Q}$ measures were time aligned with Innocor during rest and each measurement up to 160 W. Beyond 160 W, we employed a correction factor to our Finometer estimates of $\dot{Q}$ to provide corrected, more accurate measures of $\dot{Q}$ at higher exercise intensities similar to (Tam et al. 2004). Our method of correction consisted of a linear regression of Finometer estimates of $\dot{Q}$ and Innocor measures of $\dot{Q}$ up to 160 W. Once an individual's relationship was established, given an estimated Finometer measure of $\dot{Q}$, an Innocor corrected $\dot{Q}$ measure could be computed. Although individual corrections were applied, this method produced a linear relationship between Innocor measures of $\dot{Q}$ and Finometer estimates of $\dot{Q}$ (slope values of 1.1 ± 0.3) and *r* ^2^ values (0.95 ± 0.04).

#### HR and arterial blood pressure and skeletal muscle oxygenation

HR, arterial blood pressure and skeletal muscle oxygenation were computed during the same time period as $\dot{Q}$.

#### Computation of blood flow (cardiac output) redistribution

At each exercise intensity eMBF, exercising muscle venous oxygen content (C_v_O_2_) and exercising muscle StO_2_, as well as other tissue blood flow and other tissue C_v_O_2_ were computed given known measures of $\dot{Q}$, $\dot{V}O_{2}$, and C_a_O_2_. To provide an estimate of other tissue blood flow that was available for redistribution in exercise, it was assumed that resting, soon to be exercising, skeletal muscle received 5 mL/100 g per minute of $\dot{Q}$ (Saltin et al. 1998; Kalliokoski et al. 2000) and that 20 kg of muscle would participate in the cycling exercise.

It is important to understand that the muscle mass estimate was only used in estimating baseline muscle blood flow in what would be the soon to be exercising muscles. By subtracting this from $\dot{Q}$ we obtained our estimate of resting blood flow to nonexercising tissue, which would be available for redistribution. Recognizing that 20 kg might be an over estimation of the exercising muscle mass in some individuals and an underestimation in others, it is important to consider its impact on estimated other tissue blood flow at rest. An estimate of resting muscle blood flow from the reported values in the literature (Saltin et al. 1998; Kalliokoski et al. 2000) represents 50 mL/kg per minute. Therefore, even if the range of soon to be exercising muscle across individuals was as much as 10 kg, the largest differences in estimated resting blood flow of nonexercising tissue would be 500 mL/min.

In exercise, increases in $\dot{Q}$ beyond resting levels were assumed to perfuse active skeletal (Armstrong et al. 1987) and nonexercising tissue $\dot{V}O_{2}$ was assumed to remain constant. The lowest plausible exercising muscle venous saturation of 15% (Costes et al. 1996; MacDonald et al. 1999) was used as a cutoff for the requirement of blood flow redistribution from other tissue to augment $\dot{Q}$. This redistribution was only calculated for an individual at a given exercise intensity if the increase in muscle blood flow corresponding to the measured increase in $\dot{Q}$ was inadequate to maintain at least this cutoff C_v_O_2_. The required redistribution to result in the cutoff C_v_O_2_ was then calculated. Thus, the comparison of lower and higher cardiac output blood flow redistribution response reflects only the response that would be absolutely essential.

The goal of this was to establish that adequate resting tissue blood flow was available for redistribution to meet this minimum C_v_O_2_ cutoff. In other words, that redistribution was a plausible mechanism for explaining how exercising muscle oxygenation could be the same across individuals with considerably different systemic O_2_D ($\dot{Q}$). In addition, group analysis for lower and higher cardiac responders was completed in which exercising muscle C_v_O_2_ for lower cardiac responders was matched to higher cardiac responders at the group level and other tissue C_v_O_2_ was calculated for each exercise intensity given known values of $\dot{Q}$, $\dot{V}O_{2}$, and C_a_O_2_.

### Statistical analysis

**Figure 2:**
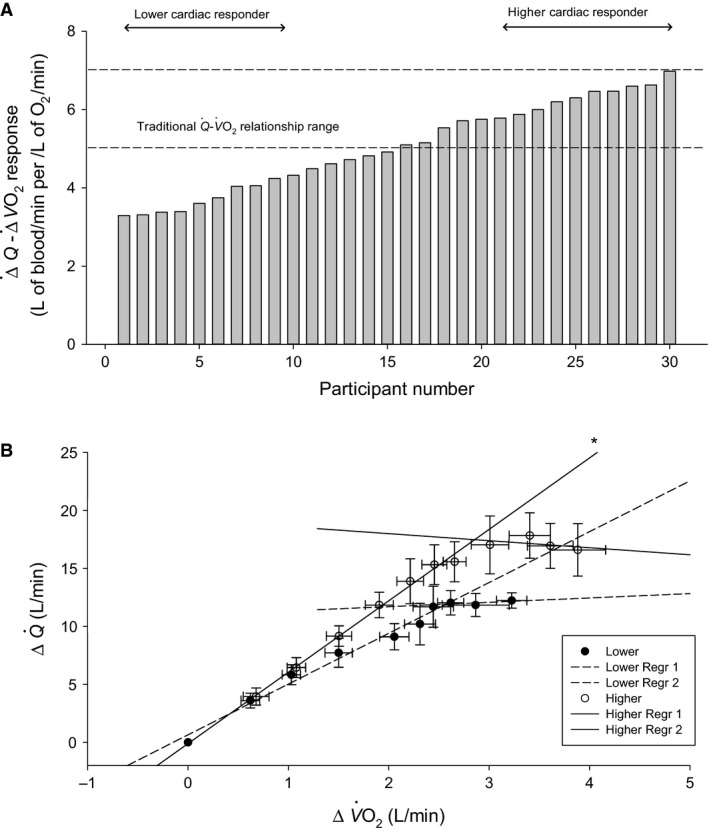
Cardiac responses to progressive exercise. (A) The increase in cardiac output ($\dot{Q}$) required for a given increase in oxygen consumption ($\dot{V}O_{2}$) from 40 to 160 W. Individual slope responses are presented from lowest to highest. Dashed lines express the traditional slope response. Lower and higher cardiac responders were identified as the lowest and highest 10 individual responses, respectively. (B) Mean $\Delta \dot{Q}$ required for a $\Delta \dot{V}O_{2}$ at each exercise intensity for lower and higher cardiac responders. Linear regressions are plotted for lower (dashed lines) and higher (solid lines) responder groups. Regressions were completed up to the plateau, and beyond the plateau in $\Delta \dot{Q}$ independently. *Statistically significant difference between lower versus higher responders *P *<* *0.05.

A linear regression was completed for each participant's $\Delta \dot{Q}$ versus $\Delta \dot{V}O_{2}$ data from 40 to 160 W to identify individual $\dot{Q}-\dot{V}O_{2}$ relationships. This exercise intensity range was used because it ensured that all participant fits were based on the same exercise intensity increments, as with increasing exercise intensity there was participant attrition beyond 160 W. Individual slope responses were ranked, and participants with the 10 lowest and 10 highest slopes were grouped post hoc as lower and higher cardiac responders, respectively (Fig. 2A). All subsequent analysis was completed on these two groups, unless otherwise noted.

Following post hoc identification of lower and higher cardiac response groups, $\Delta \dot{Q}$ was plotted against $\Delta \dot{V}O_{2}$ at each exercise intensity for each group. A linear regression was completed for each group's data, and slope analysis of these regressions was completed to determine if there was a significant between‐group difference (Fig. 2B).

A one way repeated measures mixed model ANOVA was used to compare lower versus higher cardiac responders for *Δ* $\dot{Q}$, SV, HR StO_2_, $\dot{Q}$ redistribution, exercising muscle C_V_O_2_, other tissue C_V_O_2_ at each exercise intensity from 40 to 185 W (Figs. 4–6). Additional one way repeated measures mixed model ANOVAs were completed for *Δ* mean arterial pressure (MAP), TVC, RPE and $\dot{V}O_{2}$ (data not shown). Due to variability in fitness, two lower responders did not complete the 185 W exercise intensity. Therefore, one, one way repeated measures mixed model ANOVA was completed from 40 to 160 W for all 20 participants (lower; *n* = 10 and higher; *n* = 10). Then, only participants who completed 185 W were used in a subsequent ANOVA for the remaining 18 participants (lower; *n* = 8 and higher; *n* = 10). This method was completed previously (Bentley et al. 2014) and allows us to maximize the strength of the repeated measure ANOVA.

**Figure 3:**
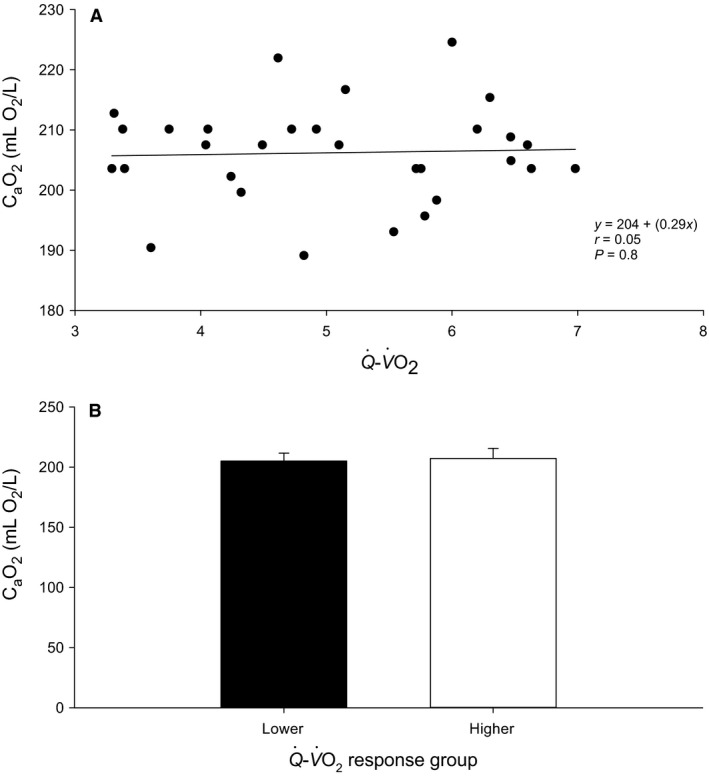
Arterial oxygen content (C_a_O_2_). (A) Correlation between $\dot{Q}-\dot{V}O_{2}$ and C_a_O_2_ with all participants. (B) Lower versus Higher cardiac response group comparison. Not significant (NS), *P* > 0.05.

Linear regressions across all participants were completed for $\dot{Q}-\dot{V}O_{2}$ versus RPE (data not shown) and *Δ*StO_2_ (Fig. 5) to confirm that an observed difference between lower and higher cardiac responders for a given variable at each exercise intensity reflected the relationship across all participants. These were conducted for each exercise intensity between 40 and 185 W in which $\Delta \dot{Q}$ was statistically significantly reduced in lower compared to higher cardiac responders (120 W; *n* = 30, 160 W; *n* = 30, 185 W; *n* = 28). Linear regressions across all participants were also completed at these intensities for individual *Δ*SV, *Δ*HR and *Δ*TVC versus $\dot{Q}$ (data not shown). Lastly, linear regressions were completed across all participants for individual $\dot{Q}-\dot{V}O_{2}$ relationships versus peak exercise intensity (data not shown), $\dot{V}O_{2}$ peak (data not shown), $\dot{Q}$ peak (data not shown) and C_a_O_2_ (Fig. 3).

Unpaired Student *t*‐tests were used to compare peak responses between lower versus higher cardiac response groups as well as baseline values, skeletal muscle properties and anthropometric data (Table 1, Fig. 3).

**Table 1:** Anthropometric measures, baseline values, peripheral skeletal muscle characteristics, peak oxygen consumption and cardiac output – oxygen consumption relationship

Variable	All (*n* = 30)	Lower (*n* = 10)	Higher (*n* = 10)
Age (years)	21 ± 3	22 ± 3	21 ± 2
Height (cm)	182 ± 7	180 ± 7	182 ± 6
Weight (kg)	76 ± 10	75 ± 12	81 ± 7
BMI	23.1 ± 2.4	23.2 ± 3.0	24.5 ± 2.1
7 day PAR score (METS/week)	248 ± 17	244 ± 13	249 ± 15
MAP_BSL_ (mmHg)	91 ± 9	93 ± 9	90 ± 8
HR_BSL_ (bpm)	84 ± 12	94 ± 11	73 ± 10^a^
SV_BSL_ (mL/beat)	75 ± 15	70 ± 20	83 ± 11
${\dot{Q}}_{\mathrm{BSL}}$ (L/min)	6.2 ± 1.1	6.4 ± 1.5	6.0 ± 1.0
$\dot{V}O_{2\mathrm{BSL}}$ (L/min)	0.34 ± 0.05	0.33 ± 0.05	0.33 ± 0.05
TVC_BSL_ (L/min per 100 mmHg)	6.8 ± 1.1	6.9 ± 1.4	6.7 ± 1.0
StO_2BSL_ (%)	71 ± 6	71 ± 3	70 ± 6
C_a_O_2_ (mL O_2_/L)	206 ± 8	205 ± 7	207 ± 8
Capillary density (mm^2^)	468 ± 85	456 ± 67	457 ± 107
Type I fiber (%)	51 ± 12	44 ± 12	51 ± 6
Type II fiber (%)	49 ± 12	56 ± 12	49 ± 6
Type I fiber SDH activity (A.U.)	38 ± 9	40 ± 9	36 ± 9
Type II fiber SDH activity (A.U.)	27 ± 9	29 ± 8	26 ± 9
$\dot{V}O_{2}$pk (L/min)	3.0 ± 0.5	2.6 ± 0.4	3.4 ± 0.5^a^
$\Delta \dot{Q}-\Delta \dot{V}O_{2}$ (L/min of blood per L/min O_2_)	5.0 ± 1.2	3.7 ± 0.4	6.3 ± 0.4^a^

Values are mean ± SD. BMI, body mass index; PAR, physical activity recall; MAP, mean arterial pressure; HR, heart rate; SV, stroke volume; $\dot{Q}$, cardiac output; TVC, total vascular conductance; StO_2_, hemoglobin saturation; C_a_O_2_, arterial oxygen content; SDH, succinate dehydrogenase; A.U., arbitrary units; $\dot{V}O_{2}$pk, peak rate of oxygen consumption; $\dot{Q}-\dot{V}O_{2}$, increase in cardiac output required per increase in oxygen consumption.

a Statistically significant difference between lower and higher cardiac responders, *P* < 0.05.

Statistical significance was set at *P* < 0.05. Only significant F‐statistics within the ANOVA for group by exercise intensity were further assessed using Bonferroni corrected post hoc tests. All assumptions of the repeated measure mixed model ANOVA were met including normality, homogeneity of regression slopes and sphericity. When the assumption of sphericity was not met, a Greenhouse‐Geisser correction was applied when determining *F*‐statistic significance. When linear regressions were completed at 120, 160, and 185 W, a Bonferroni correction was applied to the significance level to maintain family wise error rate (*P* = 0.05/3). Statistical comparisons were only completed up to 185 W due to participant attrition at higher exercise intensities. Statistics were calculated using a combination of SPSS 20 (IBM Software), SigmaPlot 12.0 (Systat Software, Inc.) and GraphPad Prism 6 (GraphPad Software, Inc.). All results presented are mean *Δ* from baseline ± SD unless otherwise noted. During the calculation of individual $\dot{Q}-\dot{V}O_{2}$ and subsequent systemic C_v_O_2_ validation, one participant had an implausible systemic C_v_O_2_ at 160 W (for more information see Results). This participant was removed from our data set and analysis was completed with an *n* = 30.

## Results

### Standard anthropometric data and baseline values

Standard anthropometric data is presented in Table 1 alongside baseline values. There was no difference between any parameters except lower cardiac responders had a higher HR_BSL_ (94 ± 11 vs. 73 ± 10, *P* < 0.001).

### Identification of higher and lower cardiac responses

**Figure 4:**
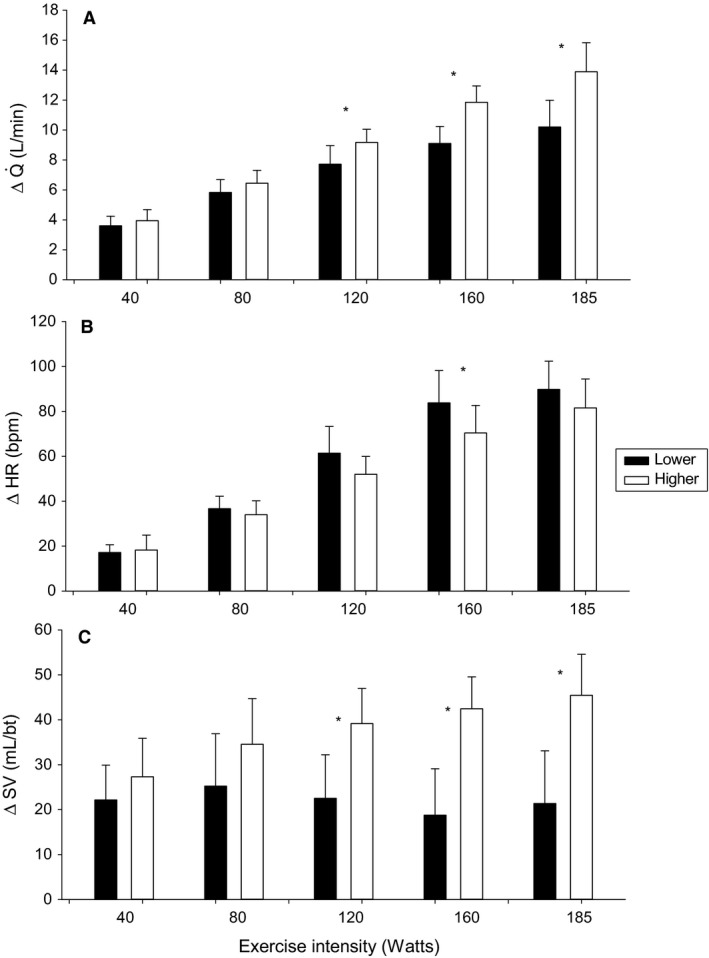
Cardiac output and constituents during progressive exercise. (A) Cardiac output ($\Delta \dot{Q}$). (B) Heart rate (*Δ*HR). (C) Stroke volume (*Δ*SV). *Statistically significant difference between lower versus higher cardiac responders *P* < 0.05.

Analysis of the $\dot{Q}-\dot{V}O_{2}$ from 40 to 160 W revealed slope responses ranging from 3.3 to 7.0 L/min of blood per L/min of oxygen (Fig. 2A). Higher and lower cardiac responders were identified post hoc (*n* = 10/group). These two groups differed in their $\dot{Q}-\dot{V}O_{2}$ relationship (6.3 ± 0.4 vs. 3.7 ± 0.4 L of blood per L of oxygen, *P* < 0.001) (Fig. 2B). $\Delta \dot{Q}$ was reduced in lower responders from 120 to 185 W (all *P* < 0.008, Fig. 4A). Computed systemic C_v_O_2_, which can be used to determine the validity in $\dot{Q}$ measures, revealed only one implausible value (0 < C_v_O_2_ < 20 mL O_2_/L) at 160 W. This participant was removed from all analysis. All other values were plausible (C_v_O_2_ ≥ 20 mL O_2_/L) (data not shown).

### Explanation of disparate cardiac responses

Observed differences in cardiac responses across all participants were not correlated with C_a_O_2_ (*r* = 0.04, *P* = 0.8; Fig. 3A). Furthermore, lower and higher cardiac response groups did not differ in C_a_O_2_ (205 ± 7 vs. 207 ± 8 mL O_2_/L blood; *P* = 0.5) (Fig. 3B). As such, differences in $\dot{Q}$ represent differences in systemic O_2_D. The presence of a varying cardiac response was not attributed to differences in skeletal muscle properties of the vastus lateralis (Table 1). Both lower and higher cardiac responders did not differ with respect to capillary density, type I and type II fiber type composition, and type I and type II fiber type specific SDH activity (all *P* > 0.1). Across all participants, there was no relationship between $\dot{Q}-\dot{V}O_{2}$ and any of these variables (*r *= 0.15–0.25, *P* = 0.4–0.2; data not shown). *Δ*MAP was not different between lower and higher cardiac responders (all *F* tests *P* > 0.2; data not shown). Contributing to the blunted $\Delta \dot{Q}$ in lower responders, *Δ*SV was less than higher responders from 120 to 185 W (all *P* < 0.001, Fig. 4C), while *Δ*HR was not different except for 160 W where it was greater (*P* = 0.04, Fig. 4B). Across all participants, *Δ*SV was significantly positively correlated with $\Delta \dot{Q}$ at 120, 160, and 185 W (all *P* < 0.001; data not shown) as was *Δ*TVC (all *P* < 0.001; data not shown). *Δ*HR was not correlated with $\Delta \dot{Q}$ (all *P* > 0.3; data not shown).

### Are interindividual differences in cardiac output associated with skeletal muscle oxygenation and perceived exertion?

**Figure 5:**
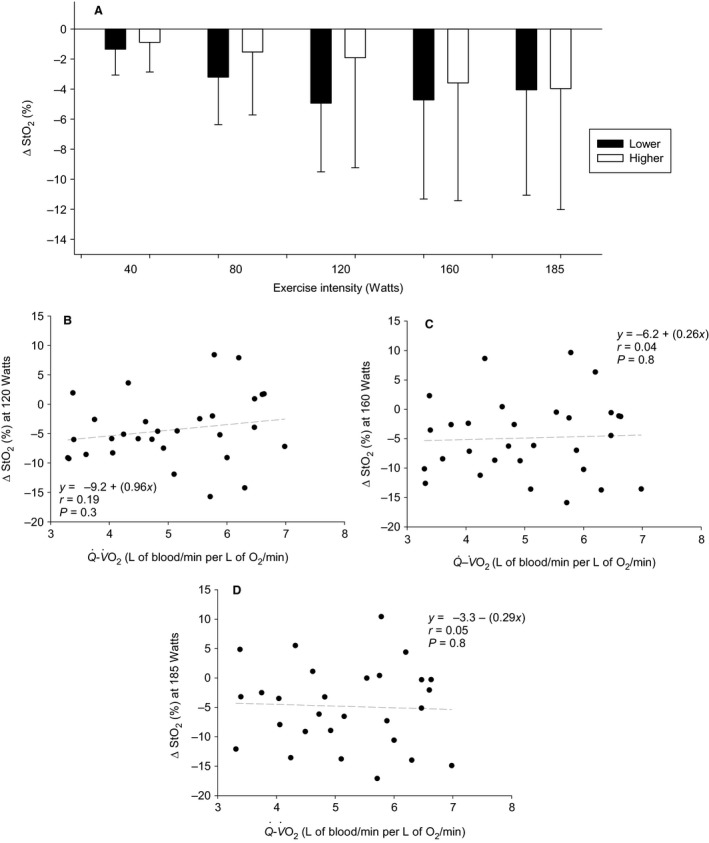
Vastus lateralis saturation (*Δ*StO_2_). (A) Lower versus Higher cardiac response group comparison. (B) Correlation between $\dot{Q}-\dot{V}O_{2}$ and vastus lateralis *Δ*StO_2_ across all participants at 120 W. (C) Correlation between $\dot{Q}-\dot{V}O_{2}$ and vastus lateralis *Δ*StO_2_ across all participants at 160 W. (D) Correlation between $\dot{Q}-\dot{V}O_{2}$ and vastus lateralis *Δ*StO_2_ across all participants at 185 W. Not significant (NS), *P* > 0.05.

Skeletal muscle (vastus lateralis) saturation (StO_2_) was not different (all *F* tests *P* > 0.4; Fig. 5A). Across all participants there was not relationship between $\dot{Q}-\dot{V}O_{2}$ and *Δ*StO_2_ at 120 W (*P* = 0.3), 160 W (*P* = 0.8) or 185 W (*P* = 0.8) (Fig. 5B–D). Lower responders had a higher RPE_L_ (17.5 ± 2.5 vs. 15.8 ± 1.5; *P* = 0.027) (data not shown) and RPE_WB_ (17 ± 2.7 vs. 15.6 ± 1.7; *P* = 0.03) at 185 W, but this was at a higher percentage of peak $\dot{V}O_{2}$ (185 W, 87 ± 15% vs. 66 ± 12%, *P* = 0.005).

The relationship between HR and RPE was similar between higher and lower cardiac responders (data not shown). Across all participants there was no relationship between $\dot{Q}-\dot{V}O_{2}$ and RPE_L_ at 120 W (*P* = 0.4), 160 W (*P* = 0.2) or 185 W (*P* = 0.2) or RPE_WB_ a 120 W (*P* = 0.7), 160 W (*P* = 0.5) or 185 W (*P* = 0.1).

**Figure 6:**
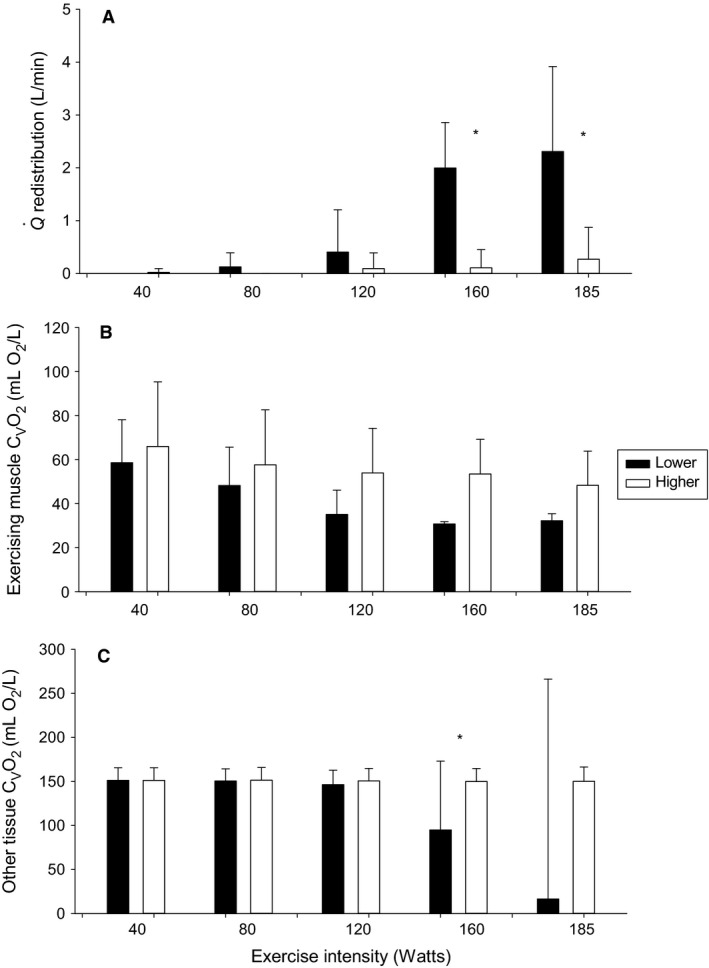
Cardiac output redistribution during exercise. (A) Redistribution required to maintain a computed minimum of 15% exercising muscle venous saturation. (B) Corresponding exercising muscle venous oxygen content (C_V_O_2_). (C) Corresponding other tissue C_V_O_2_. *Statistically significant difference between lower versus higher cardiac responders *P* < 0.05.

Individual assessment of lower cardiac responders demonstrated a greater computed $\dot{Q}$ redistribution requirement compared to higher cardiac responders at 160 W (2.0 ± 0.8 vs. 0.1 ± 0.3 L/min, *P* < 0.001) and 185 W (2.3 ± 1.6 vs. 0.3 ± 0.6 L/min, *P* = 0.003) was required in order to achieve exercising muscle C_v_O_2_ that was not different from higher responders where no redistribution was required (all *F* tests *P* > 0.3). Computed other tissue C_v_O_2_ was lower in lower responders at 160 W (95 ± 78 vs. 150 ± 15 L O_2_/min, *P* = 0.04; Fig. 6). Across all participants, exercising muscle C_v_O_2_ was matched at each exercise intensity to higher cardiac responders. This resulted in computed other tissue C_v_O_2_ that were plausible at all exercise intensities (all other tissue C_v_O_2_ > 50 mL O_2_/L; Table 2).

**Table 2:** Lower cardiac responders computed cardiac output redistribution

Exercise intensity (W)	$\dot{Q}$ Redistribution (L/min)	Exercising muscle C_v_O_2_ (mL O_2_/L)	Other tissue C_v_O_2_ (mL O_2_/L)
40	0.2	65	152
80	0.5	58	149
120	1.4	52	136
160	3.6	52	51
185	3.3	44	84

Cardiac output redistribution at the group level at each exercise intensity for lower cardiac responders and the associated other tissue C_v_O_2_ when exercising muscle C_v_O_2_ is matched to higher cardiac responders. $\dot{Q}$, cardiac output; C_v_O_2_, venous oxygen content.

### Functional impact of higher versus lower cardiac responders

Higher and lower cardiac responders had similar $\Delta \dot{V}O_{2}$ at each completed submaximal exercise intensity (all *F* tests *P* > 0.1; data not shown). Higher cardiac responders had a higher peak exercise intensity (295 ± 40 vs. 217 ± 53 W; *P* = 0.001) (data not shown), a greater peak $\Delta \dot{Q}$ (17.3 ± 2.3 vs. 11.0 ± 2.0 L/min; *P* < 0.001) (data not shown) and a greater peak $\dot{V}O_{2}$ (48.8 ± 3.9 vs. 39.2 ± 6.2 mL/min per kg; *P* = 0.004) (data not shown). There was also a significant relationship between $\dot{Q}-\dot{V}O_{2}$ and peak exercise intensity (*r* = 0.61, *P* < 0.001), peak $\Delta \dot{Q}$ (*r* = 0.63, *P* < 0.001) and peak $\dot{V}O_{2}$ (*r* = 0.5, *P* = 0.006) assessed across all participants (data not shown).

## Discussion

The primary novel findings of the study were as follows: (1) There was a considerable range in the $\dot{Q}-\dot{V}O_{2}$ relationship across individuals allowing for the identification of lower and higher cardiac responders in our sample. (2) Differences in SV accounted for $\dot{Q}-\dot{V}O_{2}$ responder group differences, not C_a_O_2_ or skeletal muscle properties. (3) Interindividual differences in $\dot{Q}-\dot{V}O_{2}$ were not associated with differences in exercising muscle oxygenation or RPE.

These findings support the hypothesis that considerable interindividual differences in the increase in $\dot{Q}$ for a given increase in $\dot{V}O_{2}$ exist among young healthy similarly active males. However, they do not support the hypothesis that individuals with lower $\dot{Q}-\dot{V}O_{2}$ would present with reduced oxygenation and increased RPE. Instead, they suggest that in lower responders, greater vasoconstriction in nonexercising tissue may compensate and thereby protect exercising muscle O_2_D.

### Interindividual differences in $\dot{Q}-\dot{V}O_{2}$

Previous studies quantifying $\dot{Q}-\dot{V}O_{2}$ at the group level generally report averages of 5–7 L of blood/min per L of O_2_/min (Faulkner et al. 1977; Proctor et al. 1998; Adami et al. 2014), although literature from the 1960s (Reeves et al. 1961; Astrand et al. 1964) and 1980s (Yamaguchi et al. 1986) have reported interindividual differences in $\dot{Q}$ as great as 5–6 L/min at the same submaximal exercise $\dot{V}O_{2}$. Summarizing within subject manipulations in C_a_O_2_ and their effects on $\dot{Q}-\dot{V}O_{2}$ (Adami et al. 2014) suggests that $\dot{Q}-\dot{V}O_{2}$ differences are thought to reflect constant systemic O_2_D under varying C_a_O_2_. To the best of our knowledge there is one study exploring interindividual differences in [Hb] and submaximal $\dot{Q}$ (Freedson 1981). This work completed in women aligns with the conclusions of (Adami et al. 2014) providing [Hb] is between ~10 and 13.5 g/100 mL blood. However, [Hb] greater than 13.5 g/100 mL blood appears unrelated to $\dot{Q}$. In this study, [Hb] ranged from 14.4 to 17.1 g/100 mL. Our findings agree with and extend the findings of (Freedson 1981) and refute the common assumptions that interindividual differences in $\dot{Q}-\dot{V}O_{2}$ are simply a reflection of compensation for differences in C_a_O_2_, and that systemic O_2_D is tightly and similarly regulated across healthy individuals.

During our progressive exercise test, we observed individual $\dot{Q}-\dot{V}O_{2}$ responses ranging from 3.3 to 7.0 L of blood/min per L of O_2_/min. This resulted in the post hoc classification of what we termed lower and higher cardiac responders, respectively. The 40–160 W exercise intensity range was used to establish $\dot{Q}-\dot{V}O_{2}$ due to the use of inert gas rebreathe and the inclusion of all participants. Computed mixed venous oxygen content at a given $\dot{V}O_{2}$ was used to identify plausible (CvO_2_ ≥ 20 mL O_2_/L), implausible (0 < C_v_O_2_ < 20 mL O_2_/L) and impossible (C_v_O_2_ < 0 mL O_2_/L) values (Siebenmann et al. 2015). This analysis revealed only one implausible value across all $\dot{Q}$ measurements from rest to 160 W. This individual was removed from the analysis. Therefore, we believe the observed presence of large interindividual differences in remaining $\dot{Q}-\dot{V}O_{2}$ can be interpreted with confidence.

The *Δ*SV was a strong predictor of $\dot{Q}-\dot{V}O_{2}$ across all individuals and was significantly lower in the lower cardiac responder group, whereas *Δ*HR was unrelated to $\dot{Q}-\dot{V}O_{2}$ and not different between groups. We could not measure specific parameters of cardiac function such as preload, ventricular filling rate, or indices of contractility so we are unable to definitively identify the reason for SV differences. However, both β‐2 adrenergic receptor polymorphisms at codon 16 (Arginine/Arginine vs. Glycine/Glycine) (Snyder et al. 2006a) and density of cardiac β‐2 adrenergic receptors (Snyder et al. 2006b) have been linked to interindividual differences in SV at the same $\dot{V}O_{2}$, likely due to β‐2 adrenergic receptors contribution to contractility. Similar to our findings, differences in SV observed in these studies were not offset by HR and therefore the $\dot{Q}$ response to exercise was blunted with a lower SV.

It has also been demonstrated that an acute increase in blood volume leads to increased SV in submaximal exercise (Kanstrup and Ekblom 1982; Hopper et al. 1988; Krip et al. 1997), although statistically significant increases in $\dot{Q}$ were only observed by (Krip et al. 1997). Measured effects of blood volume on maximal ventricular filling rate (Krip et al. 1997) in exercise suggest the potential for blood volume dependent pre‐load differences influencing diastolic filling properties as another possible explanation for our findings. Lastly, differences in left ventricular volume determining the peak filling capacity (and therefore the limits of SV) cannot be ruled out. In summary, whether contractility, blood volume‐mediated cardiac filling, ventricular volume, or all three explain our findings warrant future investigation.

### Skeletal muscle oxygenation and RPE

In this study, we utilized NIRS to quantify skeletal muscle oxygenation. NIRS is a surrogate measure of O_2_D: demand matching as oxygenation represents the balance between blood flow into the exchange vessels and extraction of oxygen by the tissues. Therefore, at the same $\dot{V}O_{2}$ greater oxygenation reflects relatively greater perfusion. We observed *Δ*StO_2_ values ranging from −1% to −7%. This magnitude of change aligns with previous cycling exercise studies (Okushima et al. 2015; Hirai et al. 2017).

Contrary to our hypothesis, $\dot{Q}-\dot{V}O_{2}$ was not related to exercising muscle oxygenation at any exercise intensity whether assessed across all participants or compared between responder groups. Consistent with this was the absence of a relationship between $\dot{Q}-\dot{V}O_{2}$ and RPE regardless of exercise intensity when assessed across all participants. While analysis of lower versus higher responder groups revealed an elevated RPE_L_ and RPE_WB_ in lower cardiac responders at 185 W, this represented a higher %$\dot{V}O_{2}$ peak in lower versus higher responders (87 ± 15% vs. 66 ± 12%, *P* = 0.005) and this difference likely explains the RPE difference. This interpretation was confirmed across all participants as correlational analysis revealed that both RPE_L_ and RPE_WB_ at 185 W were significantly negatively correlated with $\dot{V}O_{2}$ peak (*r* = −0.5, *P* = 0.003 and *r* = −0.7, *P* < 0.001, respectively).

### Implications for co‐ordinated blood pressure and oxygen delivery regulation

The lack of difference between responder groups in *Δ*StO_2_ and *Δ*MAP indicates that the exercising muscle vasodilation response for a given $\dot{V}O_{2}$ was similar. However, lower responders had a significantly lower *Δ*TVC with increasing exercise intensity, and the *Δ*TVC was also strongly correlated with $\Delta \dot{Q}$. Taken together, our findings suggest lower and higher cardiac responder groups differ in neural control determining the balance of central cardiac and nonexercising tissue vascular responses in defense of arterial blood pressure and exercising muscle O_2_D: demand matching (Nobrega et al. 2014; Michelini et al. 2015). Specifically, our findings suggest greater vasoconstriction in nonexercising skeletal muscle and other tissues (Armstrong et al. 1987; Mortensen et al. 2008; Calbet et al. 2015) in order to regulate arterial blood pressure in individuals with a lower $\dot{Q}-\dot{V}O_{2}$.

This between‐subject explanation aligns with previous within‐subject work by (Pawelczyk et al. 1992) during submaximal cycling exercise. These investigators found that a reduction in submaximal exercise $\dot{Q}$ via cardio‐selective β_1_ adrenergic receptor blockade did not result in a statistically significant decrease in exercising leg vascular conductance, except at the highest work rate, where it represented only ~35% of the reduction in systemic vascular conductance. Thus, substantial vasoconstriction in nonexercising tissue was the primary compensation for reduced $\dot{Q}$ as part of combined arterial blood pressure regulation and exercising muscle O_2_D: demand matching in submaximal exercise.

To assess the feasibility of redistribution explaining the findings in our study we estimated the degree of $\dot{Q}$ redistribution at each exercise intensity with the constraint that the exercising muscle StO_2_ could not go below 15% (Costes et al. 1996; MacDonald et al. 1999). Individually, during exercise up to 185 W in lower cardiac responders, a total of 24 out of 47 measures required $\dot{Q}$ redistribution to maintain exercising muscle StO_2_ at 15% while 7 out of 50 measures in higher cardiac responders required $\dot{Q}$ redistribution. In lower responders, only 3 out of 24 redistribution calculations resulted in an impossible level of $\dot{Q}$ redistribution (with one participant heavily influencing the mean of other tissue C_v_O_2_ at 185 W in Fig. 6C), while all higher cardiac responder redistributions were plausible. At the group level, we matched exercising muscle C_v_O_2_ in lower cardiac responders to higher cardiac responders at each exercise intensity. We then computed the $\dot{Q}$ redistribution and other tissue C_v_O_2_ required to support this level of exercising muscle C_v_O_2_. This resulted in plausible other tissue C_v_O_2_ up to 185 W with an average of 3.6 and 3.3 L/min of available $\dot{Q}$ being redistributed at 160 and 185 W, respectively (Table 2). This analysis supports the plausibility of exercising muscle saturation being maintained in lower cardiac responders with $\dot{Q}$ redistribution.

Given that arterial blood pressure is a product of $\dot{Q}$ and TVC, the question arises as to the origin of these interindividual differences between lower and higher cardiac responders. Does a reduced $\dot{Q}$ necessitate increased reflex vasoconstriction of nonexercising tissue, or does increased vasoconstriction constrain $\dot{Q}$? The former would be expected if, at the onset of an increased exercise intensity, a blunted SV response led to a greater imbalance between $\Delta \dot{Q}$ and *Δ* peripheral blood flow. The resulting compromise to arterial blood pressure would serve as a greater stimulus for baroreflex‐mediated vasoconstriction, with functional sympatholysis protecting exercising muscle O_2_D (Tschakovsky and Hughson 2003). $\dot{Q}$ and total peripheral blood flow would then stabilize at a lower level for the same arterial blood pressure while exercising muscle perfusion would be preserved. Whether the magnitude of increase in $\dot{Q}$ at a given instant in the adjustment phase to a new steady state determines such a peripheral vasoconstrictor response remains to be determined.

The latter would be expected if an inherently greater increase in sympathetic vasoconstriction was initiated with exercise in the lower cardiac responder, such that it blunted the increase in venous return. The importance of peripheral dilation as a determinant of cardiac function via venous return effects on SV has been demonstrated by (Bada et al. 2012) who observed that cardiac pacing per se cannot elevate $\dot{Q}$, whereas vasodilator infusion in a resting limb leads to large increases in limb blood flow and concomitant increases in $\dot{Q}$ in large part due to elevated SV. It is therefore plausible that greater early sympathetic vasoconstrictor response to exercise in lower versus higher responders could blunt venous return from nonexercising tissue, thereby restricting the increase in $\dot{Q}$.

In summary, it is difficult to ascertain whether a blunted $\dot{Q}$ via cardiocentric SV limitations (i.e., not related to peripheral vasodilation‐mediated venous return) leads to greater vasoconstriction, or whether greater non‐exercising tissue vasoconstriction leads to a blunted $\dot{Q}$. Thus, it is clear that there is a need for further research to clarify cardiocentric versus peripherocentric driven determinants of $\dot{Q}$ in exercise and their role in interindividual differences in $\dot{Q}-\dot{V}O_{2}$. Investigating temporal aspects of the integrated responses during the transition to a new steady state would seem critical in this regard.

### Experimental considerations

In the present investigation there are a number of strengths that provide confidence in our observations and subsequent data interpretations. The strengths of this study include: (1) The reproducibility of the inert gas rebreathe technique in our hands, which allows confidence that interindividual differences in measured $\dot{Q}$ are real. (2) The comprehensive evaluation of central and peripheral characteristics of circulation and skeletal muscle characteristics to provide an integrated picture of the underlying physiological response. (3) The use of four exercise intensities to improve confidence in slope parameter estimation of individual $\dot{Q}-\dot{V}O_{2}$.

However, there are potential limitations that need to be acknowledged. First, underestimation of $\dot{Q}$ can occur with the inert gas rebreathe technique if the rebreathe period extends into the time it takes for the insoluble gas to recirculate into the pulmonary gas exchange site. This recirculation can vary depending on size of an individual (shorter for a smaller individual) and the $\dot{V}O_{2}$ (shorter for a higher $\dot{V}O_{2}$) (Becklake et al. 1962; Zeidifard et al. 1976; Cumming 1978). At a $\dot{V}O_{2}$ of 3 L/min, recirculation has been observed to occur in as little as 8.5 sec (Rigatto et al. 1968; Sowton et al. 1968). We conducted rebreathe measurements in which the participant was coached to maintain their normal breathing frequency and tidal volume as hyperventilation has been shown to elevate $\dot{Q}$ (Donevan et al. 1962; Cummin et al. 1986). In our model, at 160 W Innocor rebreathing time was 12.5 ± 1.9 sec. However, $\dot{V}O_{2}$ was ~2.3 L/min and therefore recirculation was likely significantly slower than in the above cited work. Most importantly there was no difference between groups in rebreathe duration at any exercise intensity (all *P* > 0.5; data not shown), nor was there a difference in body size. Therefore, it is highly unlikely that recirculation explains differences in $\dot{Q}-\dot{V}O_{2}$ between lower and higher cardiac responders in our healthy population without ventilation‐perfusion abnormalities.

Second, NIRS provides an indirect assessment of perfusion relative to metabolic demand and may underestimate the magnitude of venous extraction (Costes et al. 1996; MacDonald et al. 1999; Esaki et al. 2005). However, such underestimation would be systematic and therefore contribute equally to both responder groups. It is also important to note the potential implications of fat mass, influence of blood volume changes and the influence of skin blood flow during prolonged exercise (for review see, Ferrari et al. 2011). In the present study, lower and higher cardiac responders did not differ with respect to body mass index and presumably fat mass at the measurement site, and exercise duration to 185 W was performed in a temperature controlled room so systematic group differences in skin blood flow are unlikely.

Third, RPE is a psycho‐physiological manifestation of exercise stress. As it is a self‐reported measurement, this subjectivity may mean it can vary between individuals independent of actual physiological manifestations of exercise. However, at the same time real life exercise tolerance is better reflected by RPE, as the decision to reduce or stop exercising at a certain intensity is based on how one “feels.” Furthermore, RPE has been shown to be directly influenced by O_2_D in exercise (Eiken and Bjurstedt 1987; Eves et al. 2002; MacNutt et al. 2015) supporting its sensitivity to the parameter investigated in this study. To ensure minimal interindividual variability in the use of RPE, we read all participants the same script explaining how they were to assess their RPE both within the leg in isolation and as a whole body assessment.

Finally, inability to assess specific parameters of cardiac function limit definitive identification of the mechanism of reduced SV, and therefore $\dot{Q}$, in the lower responder versus higher responder groups. However, this study was not designed for that purpose and provides the first indication of important interindividual differences in the balance between central and peripheral responses to exercise.

## Conclusion

During submaximal exercise we observed differences in $\dot{Q}-\dot{V}O_{2}$ between individuals of up to ~4 L of blood/min per L of O_2_/min in healthy, young, recreationally active males, allowing post hoc classification of individuals into lower and higher cardiac responders. These groups did not differ with respect to C_a_O_2_, peripheral skeletal muscle characteristics and, contrary to our hypothesis, exercising skeletal muscle oxygenation. Across all participants there was no relationship between $\dot{Q}-\dot{V}O_{2}$ and RPE. These findings are consistent with a greater degree of non‐exercising tissue vasoconstriction in lower responders which acts to redistribute blood flow to the exercising muscle in order to support similar eMBF at a lower $\dot{Q}$. A blunted *Δ*SV was associated with a reduced $\Delta \dot{Q}$ in lower versus higher cardiac responders. Careful investigation of the integrated cardiovascular response during transition to steady state will be critical for determining whether submaximal $\dot{Q}-\dot{V}O_{2}$ differences between individuals are driven by a cardiocentric or a peripherocentric effect.

## Conflict of Interest

The authors declare that they have no conflicts of interest with the contents of this article.

## References

[phy213570-bib-0001] Abbiss, C. R. , J. J. Peiffer , R. Meeusen , and S. Skorski . 2015 Role of ratings of perceived exertion during self‐paced exercise: what are we actually measuring? Sports Med. 45:1235–1243. 26054383 10.1007/s40279-015-0344-5

[phy213570-bib-0002] Adami, A. , N. Fagoni , and G. Ferretti . 2014 The *Q* ‐*V* O_2_ diagram: an analytical interpretation of oxygen transport in arterial blood during exercise in humans. Respir. Physiol. Neurobiol. 193:55–61. 24440436 10.1016/j.resp.2014.01.007

[phy213570-bib-0003] Agostoni, P. , G. Cattadori , A. Apostolo , M. Contini , P. Palermo , G. Marenzi , et al. 2005 Noninvasive measurement of cardiac output during exercise by inert gas rebreathing technique: a new tool for heart failure evaluation. J. Am. Coll. Cardiol. 46:1779–1781. 16256885 10.1016/j.jacc.2005.08.005

[phy213570-bib-0004] Armstrong, R. B. , M. D. Delp , E. F. Goljan , and M. H. Laughlin . 1987 Distribution of blood flow in muscles of miniature swine during exercise. J. Appl. Physiol. (1985) 62:1285–1298. 3106313 10.1152/jappl.1987.62.3.1285

[phy213570-bib-0005] Astrand, P. O. , T. E. Cuddy , B. Saltin , and J. Stenberg . 1964 Cardiac output during submaximal and maximal work. J. Appl. Physiol. 19:268–274. 14155294 10.1152/jappl.1964.19.2.268

[phy213570-bib-0006] Bada, A. A. , J. H. Svendsen , N. H. Secher , B. Saltin , and S. P. Mortensen . 2012 Peripheral vasodilatation determines cardiac output in exercising humans: insight from atrial pacing. J. Physiol. 590:2051–2060. 22351638 10.1113/jphysiol.2011.225334 PMC3573320

[phy213570-bib-0007] Becklake, M. R. , C. J. Varvis , L. D. Pengelly , S. Kenning , G. M. Mc , and D. V. Bates . 1962 Measurement of pulmonary blood flow during exercise using nitrous oxide. J. Appl. Physiol. 17:579–586. 13866419 10.1152/jappl.1962.17.4.579

[phy213570-bib-0008] Bentley, R. F. , J. M. Kellawan , J. S. Moynes , V. J. Poitras , J. J. Walsh , and M. E. Tschakovsky . 2014 Individual susceptibility to hypoperfusion and reductions in exercise performance when perfusion pressure is reduced: evidence for vasodilator phenotypes. J. Appl. Physiol. (1985) 117:392–405. 24970851 10.1152/japplphysiol.01155.2013 PMC4137234

[phy213570-bib-0009] Bergstrom, J. 1975 Percutaneous needle biopsy of skeletal muscle in physiological and clinical research. Scand. J. Clin. Lab. Invest. 35:609–616. 1108172

[phy213570-bib-0010] Bloemberg, D. , and J. Quadrilatero . 2012 Rapid determination of myosin heavy chain expression in rat, mouse, and human skeletal muscle using multicolor immunofluorescence analysis. PLoS One 7:e35273. 22530000 10.1371/journal.pone.0035273 PMC3329435

[phy213570-bib-0011] Borg, G. 1970 Perceived exertion as an indicator of somatic stress. Scand. J. Rehabil. Med. 2:92–98. 5523831

[phy213570-bib-0012] Brodal, P. , F. Ingjer , and L. Hermansen . 1977 Capillary supply of skeletal muscle fibers in untrained and endurance‐trained men. Am. J. Physiol. 232:H705–H712. 879309 10.1152/ajpheart.1977.232.6.H705

[phy213570-bib-0013] Calbet, J. A. , J. Gonzalez‐Alonso , J. W. Helge , H. Sondergaard , T. Munch‐Andersen , B. Saltin , et al. 2015 Central and peripheral hemodynamics in exercising humans: leg vs arm exercise. Scand. J. Med. Sci. Sports 25(Suppl. 4):144–157. 26589128 10.1111/sms.12604

[phy213570-bib-0014] Costes, F. , J. C. Barthelemy , L. Feasson , T. Busso , A. Geyssant , and C. Denis . 1996 Comparison of muscle near‐infrared spectroscopy and femoral blood gases during steady‐state exercise in humans. J. Appl. Physiol. (1985) 80:1345–1350. 8926265 10.1152/jappl.1996.80.4.1345

[phy213570-bib-0015] Cummin, A. R. , V. I. Iyawe , N. Mehta , and K. B. Saunders . 1986 Ventilation and cardiac output during the onset of exercise, and during voluntary hyperventilation, in humans. J. Physiol. 370:567–583. 3083100 10.1113/jphysiol.1986.sp015951 PMC1192697

[phy213570-bib-0016] Cumming, G. R. 1978 Recirculation times in exercising children. J. Appl. Physiol. Respir. Environ. Exerc. Physiol. 45:1005–1008. 730581 10.1152/jappl.1978.45.6.1005

[phy213570-bib-0017] Donevan, R. E. , N. M. Anderson , P. Sekelj , O. Papp , and G. M. Mc . 1962 Influence of vuluntary hyperventilation on cardiac output. J. Appl. Physiol. 17:487–491. 13887210 10.1152/jappl.1962.17.3.487

[phy213570-bib-0018] Eiken, O. , and H. Bjurstedt . 1987 Dynamic exercise in man as influenced by experimental restriction of blood flow in the working muscles. Acta Physiol. Scand. 131:339–345. 3425345 10.1111/j.1748-1716.1987.tb08248.x

[phy213570-bib-0019] Esaki, K. , T. Hamaoka , G. Radegran , R. Boushel , J. Hansen , T. Katsumura , et al. 2005 Association between regional quadriceps oxygenation and blood oxygen saturation during normoxic one‐legged dynamic knee extension. Eur. J. Appl. Physiol. 95:361–370. 16096839 10.1007/s00421-005-0008-5

[phy213570-bib-0020] Eves, N. D. , S. R. Petersen , and R. L. Jones . 2002 The effect of hyperoxia on submaximal exercise with the self‐contained breathing apparatus. Ergonomics 45:840–849. 12487686 10.1080/00140130210159995

[phy213570-bib-0021] Faulkner, J. , and R. Eston . 2007 Overall and peripheral ratings of perceived exertion during a graded exercise test to volitional exhaustion in individuals of high and low fitness. Eur. J. Appl. Physiol. 101:613–620. 17694318 10.1007/s00421-007-0536-2

[phy213570-bib-0022] Faulkner, J. A. , G. J. Heigenhauser , and M. A. Schork . 1977 The cardiac output–oxygen uptake relationship of men during graded bicycle ergometry. Med. Sci. Sports 9:148–154. 593076

[phy213570-bib-0023] Ferrari, M. , M. Muthalib , and V. Quaresima . 2011 The use of near‐infrared spectroscopy in understanding skeletal muscle physiology: recent developments. Philos. Trans. A Math. Phys. Eng. Sci. 369:4577–4590. 22006907 10.1098/rsta.2011.0230

[phy213570-bib-0024] Fontana, P. , U. Boutellier , and M. Toigo . 2009 Reliability of measurements with Innocor during exercise. Int. J. Sports Med. 30:747–753. 19642059 10.1055/s-0029-1225340

[phy213570-bib-0025] Freedson, P. S. 1981 The influence of hemoglobin concentration on exercise cardiac output. Int. J. Sports Med. 2:81–86. 7333744 10.1055/s-2008-1034587

[phy213570-bib-0026] Gratton, E. , S. Fantini , M. A. Franceschini , G. Gratton , and M. Fabiani . 1997 Measurements of scattering and absorption changes in muscle and brain. Philos. Trans. R. Soc. Lond. B Biol. Sci. 352:727–735. 9232861 10.1098/rstb.1997.0055 PMC1691951

[phy213570-bib-0027] Hepple, R. T. 2002 The role of O_2_ supply in muscle fatigue. Can. J. Appl. Physiol. 27:56–69. 11880691 10.1139/h02-004

[phy213570-bib-0028] Hirai, D. M. , J. T. Zelt , J. H. Jones , L. G. Castanhas , R. F. Bentley , W. Earle , et al. 2017 Dietary nitrate supplementation and exercise tolerance in patients with heart failure with reduced ejection fraction. Am. J. Physiol. Regul. Integr. Comp. Physiol. 312:R13–R22. 27784687 10.1152/ajpregu.00263.2016

[phy213570-bib-0029] Hopper, M. K. , A. R. Coggan , and E. F. Coyle . 1988 Exercise stroke volume relative to plasma‐volume expansion. J. Appl. Physiol. (1985) 64:404–408. 2451658 10.1152/jappl.1988.64.1.404

[phy213570-bib-0030] Kalliokoski, K. K. , J. Kemppainen , K. Larmola , T. O. Takala , P. Peltoniemi , A. Oksanen , et al. 2000 Muscle blood flow and flow heterogeneity during exercise studied with positron emission tomography in humans. Eur. J. Appl. Physiol. 83:395–401. 11138581 10.1007/s004210000267

[phy213570-bib-0031] Kanstrup, I. L. , and B. Ekblom . 1982 Acute hypervolemia, cardiac performance, and aerobic power during exercise. J. Appl. Physiol. Respir. Environ. Exerc. Physiol. 52:1186–1191. 7096143 10.1152/jappl.1982.52.5.1186

[phy213570-bib-0032] Krip, B. , N. Gledhill , V. Jamnik , and D. Warburton . 1997 Effect of alterations in blood volume on cardiac function during maximal exercise. Med. Sci. Sports Exerc. 29:1469–1476. 9372484 10.1097/00005768-199711000-00013

[phy213570-bib-0033] Larsen, S. , J. Nielsen , C. N. Hansen , L. B. Nielsen , F. Wibrand , N. Stride , et al. 2012 Biomarkers of mitochondrial content in skeletal muscle of healthy young human subjects. J. Physiol. 590:3349–3360. 22586215 10.1113/jphysiol.2012.230185 PMC3459047

[phy213570-bib-0034] MacDonald, M. J. , M. A. Tarnopolsky , H. J. Green , and R. L. Hughson . 1999 Comparison of femoral blood gases and muscle near‐infrared spectroscopy at exercise onset in humans. J. Appl. Physiol. (1985) 86:687–693. 9931209 10.1152/jappl.1999.86.2.687

[phy213570-bib-0035] MacNutt, M. J. , C. M. Peters , C. Chan , J. Moore , S. Shum , and A. W. Sheel . 2015 Day‐to‐day variability in cardiorespiratory responses to hypoxic cycle exercise. Appl. Physiol. Nutr. Metab. 40:155–161. 25603431 10.1139/apnm-2014-0297

[phy213570-bib-0036] Michelini, L. C. , D. S. O'Leary , P. B. Raven , and A. C. Nobrega . 2015 Neural control of circulation and exercise: a translational approach disclosing interactions between central command, arterial baroreflex, and muscle metaboreflex. Am. J. Physiol. Heart Circ. Physiol. 309:H381–H392. 26024683 10.1152/ajpheart.00077.2015 PMC4631530

[phy213570-bib-0037] Mortensen, S. P. , R. Damsgaard , E. A. Dawson , N. H. Secher , and J. Gonzalez‐Alonso . 2008 Restrictions in systemic and locomotor skeletal muscle perfusion, oxygen supply and *V*O_2_ during high‐intensity whole‐body exercise in humans. J. Physiol. 586:2621–2635. 18372307 10.1113/jphysiol.2007.149401 PMC2464345

[phy213570-bib-0038] Nobrega, A. C. , D. O'Leary , B. M. Silva , E. Marongiu , M. F. Piepoli , and A. Crisafulli . 2014 Neural regulation of cardiovascular response to exercise: role of central command and peripheral afferents. Biomed. Res. Int. 2014:478965. 24818143 10.1155/2014/478965 PMC4000959

[phy213570-bib-0039] Okushima, D. , D. C. Poole , H. B. Rossiter , T. J. Barstow , N. Kondo , E. Ohmae , et al. 2015 Muscle deoxygenation in the quadriceps during ramp incremental cycling: deep vs. superficial heterogeneity. J. Appl. Physiol. (1985) 119:1313–1319. 26404619 10.1152/japplphysiol.00574.2015

[phy213570-bib-0040] Pawelczyk, J. A. , B. Hanel , R. A. Pawelczyk , J. Warberg , and N. H. Secher . 1992 Leg vasoconstriction during dynamic exercise with reduced cardiac output. J. Appl. Physiol. (1985) 73:1838–1846. 1474060 10.1152/jappl.1992.73.5.1838

[phy213570-bib-0041] Peyton, P. J. , and B. Thompson . 2004 Agreement of an inert gas rebreathing device with thermodilution and the direct oxygen Fick method in measurement of pulmonary blood flow. J. Clin. Monit. Comput. 18:373–378. 15957629 10.1007/s10877-005-1589-6

[phy213570-bib-0042] Proctor, D. N. , K. C. Beck , P. H. Shen , T. J. Eickhoff , J. R. Halliwill , and M. J. Joyner . 1998 Influence of age and gender on cardiac output‐*V*O_2_ relationships during submaximal cycle ergometry. J. Appl. Physiol. (1985) 84:599–605. 9475871 10.1152/jappl.1998.84.2.599

[phy213570-bib-0043] Reeves, J. T. , R. F. Grover , S. G. Blount , and G. F. Filley, Jr . 1961 Cardiac output response to standing and treadmill walking. J. Appl. Physiol. 16:283–288. 13740332 10.1152/jappl.1961.16.2.283

[phy213570-bib-0044] Rigatto, M. , N. L. Jones , and E. J. Campbell . 1968 Pulmonary recirculation time: influence of posture and exercise. Clin. Sci. 35:183–195. 5721227

[phy213570-bib-0045] Roach, R. C. , M. D. Koskolou , J. A. Calbet , and B. Saltin . 1999 Arterial O_2_ content and tension in regulation of cardiac output and leg blood flow during exercise in humans. Am. J. Physiol. 276:H438–H445. 9950843 10.1152/ajpheart.1999.276.2.H438

[phy213570-bib-0046] Saltin, B. , G. Radegran , M. D. Koskolou , and R. C. Roach . 1998 Skeletal muscle blood flow in humans and its regulation during exercise. Acta Physiol. Scand. 162:421–436. 9578388 10.1046/j.1365-201X.1998.0293e.x

[phy213570-bib-0047] Sarkin, J. , J. Campbell , L. Gross , J. Roby , S. Bazzo , J. Sallis , et al. 1997 Project GRAD seven‐day physical activity recall interviewer's manual. Med. Sci. Sports Exerc. 29:S91–S102.

[phy213570-bib-0048] Scribbans, T. D. , B. A. Edgett , K. Vorobej , A. S. Mitchell , S. D. Joanisse , J. B. Matusiak , et al. 2014 Fibre‐specific responses to endurance and low volume high intensity interval training: striking similarities in acute and chronic adaptation. PLoS One 9:e98119. 24901767 10.1371/journal.pone.0098119 PMC4047011

[phy213570-bib-0049] Siebenmann, C. , P. Rasmussen , H. Sorensen , M. Zaar , M. Hvidtfeldt , A. Pichon , et al. 2015 Cardiac output during exercise: a comparison of four methods. Scand. J. Med. Sci. Sports 25:e20–e27. 24646113 10.1111/sms.12201

[phy213570-bib-0050] Snyder, E. M. , K. C. Beck , N. M. Dietz , J. H. Eisenach , M. J. Joyner , S. T. Turner , et al. 2006a Arg16Gly polymorphism of the beta2‐adrenergic receptor is associated with differences in cardiovascular function at rest and during exercise in humans. J. Physiol. 571:121–130. 16339181 10.1113/jphysiol.2005.098558 PMC1805638

[phy213570-bib-0051] Snyder, E. M. , M. L. Hulsebus , S. T. Turner , M. J. Joyner , and B. D. Johnson . 2006b Genotype related differences in beta2 adrenergic receptor density and cardiac function. Med. Sci. Sports Exerc. 38:882–886. 16672841 10.1249/01.mss.0000218144.02831.f6

[phy213570-bib-0052] Sowton, E. , D. Bloomfield , N. L. Jones , B. E. Higgs , and E. J. Campbell . 1968 Recirculation time during exercise. Cardiovasc. Res. 2:341–345. 4882441 10.1093/cvr/2.4.341

[phy213570-bib-0053] Tam, E. , M. Azabji Kenfack , M. Cautero , F. Lador , G. Antonutto , P. E. di Prampero , et al. 2004 Correction of cardiac output obtained by Modelflow from finger pulse pressure profiles with a respiratory method in humans. Clin. Sci. (Lond.) 106:371–376. 14606953 10.1042/CS20030302

[phy213570-bib-0054] Tschakovsky, M. E. , and R. L. Hughson . 2003 Rapid blunting of sympathetic vasoconstriction in the human forearm at the onset of exercise. J. Appl. Physiol. (1985) 94:1785–1792. 12524374 10.1152/japplphysiol.00680.2002

[phy213570-bib-0055] Yamaguchi, I. , E. Komatsu , and K. Miyazawa . 1986 Intersubject variability in cardiac output‐O_2_ uptake relation of men during exercise. J. Appl. Physiol. (1985) 61:2168–2174. 3804923 10.1152/jappl.1986.61.6.2168

[phy213570-bib-0056] Zeidifard, E. , S. Godfrey , and E. E. Davies . 1976 Estimation of cardiac output by an N_2_O rebreathing method in adults and children. J. Appl. Physiol. 41:433–438. 965316 10.1152/jappl.1976.41.3.433

[phy213570-bib-0057] Zoladz, J. A. , D. Semik , B. Zawadowska , J. Majerczak , J. Karasinski , L. Kolodziejski , et al. 2005 Capillary density and capillary‐to‐fibre ratio in vastus lateralis muscle of untrained and trained men. Folia Histochem. Cytobiol. 43:11–17. 15871557

[phy213570-bib-0058] Zurlo, F. , P. M. Nemeth , R. M. Choksi , S. Sesodia , and E. Ravussin . 1994 Whole‐body energy metabolism and skeletal muscle biochemical characteristics. Metabolism 43:481–486. 8159108 10.1016/0026-0495(94)90081-7

